# The potential pathological role of NLRP3 inflammasome activation in pediatric tic disorders and its therapeutic implications

**DOI:** 10.3389/fmed.2026.1968624

**Published:** 2026-10-23

**Authors:** Siyu Zhang, Zhuoyu Meng, Huiyang Shi, Chushi Zhang

**Affiliations:** 1 The Second Clinical College, Liaoning University of Traditional Chinese Medicine, Shenyang, China; 2 The Second Affiliated Hospital of Liaoning University of Traditional Chinese Medicine, Shenyang, China

**Keywords:** microglia, neuroinflammation, NLRP3 inflammasome, tic disorders, tourette syndrome

## Abstract

Tic disorders (TDs) are common neurodevelopmental disorders in children and adolescents, with pathophysiology involving abnormalities in cortico-striato-thalamo-cortical (CSTC) circuits, neurotransmitter imbalance, and neuroimmune dysregulation. In recent years, the NOD-like receptor family pyrin domain-containing 3 (NLRP3) inflammasome has attracted increasing attention because of its role in linking cellular stress, innate immunity, and inflammatory responses. Current human TD/Tourette syndrome (TS) studies primarily support striatal neuroimmune abnormalities and microglial inflammatory changes, whereas more direct evidence for NLRP3-, caspase-1-, and gasdermin D (GSDMD)-related alterations is derived mainly from animal models. Mechanistic evidence that NLRP3-related inflammation can influence synaptic function and neural circuits largely comes from non-TD experimental models. This review summarizes the activation mechanisms and biological effects of the NLRP3 inflammasome, with particular emphasis on its potential links to striatal neuroinflammation, pyroptosis-related effects, neurotransmitter regulation, and CSTC network abnormalities. We also discuss the pharmacological feasibility and evidentiary limitations of upstream modulation, direct NLRP3 inhibition, and blockade of downstream inflammatory effectors. At present, disease-specific causal validation of NLRP3 and direct evidence for NLRP3-targeted interventions in pediatric TDs remain lacking. Future studies integrating disease-specific mechanistic validation, biomarker development, and patient stratification are needed to clarify the pathological relevance and potential translational significance of NLRP3 in pediatric TDs.

## 1 Introduction

TDs are a group of neurodevelopmental disorders that predominantly emerge during childhood and adolescence and are characterized by recurrent, sudden, rapid, and nonrhythmic motor and/or vocal tics. TS represents the prototypical form of TDs, with a substantially higher risk in males than in females (1). The global prevalence of TS among children and adolescents is approximately 0.7% (2). The pathophysiology of TDs is complex, with core abnormalities involving impaired motor selection and inhibitory control within CSTC circuits (3), together with enhanced striatal dopaminergic signaling and dysregulation of neurotransmitter systems, including GABA and glutamate (4). Recent functional magnetic resonance imaging studies have further identified aberrant connectivity within the sensorimotor and frontoparietal control networks in medication-naïve children with TS (5). Nevertheless, conventional neurotransmitter- and circuit-based models alone do not fully explain the marked clinical heterogeneity and symptom fluctuations observed in TDs. In recent years, increasing attention has therefore been directed toward immune dysregulation, neuroinflammation, and microglial abnormalities (6). Notably, a 2025 single-nucleus transcriptomic and chromatin accessibility analysis of the caudate and putamen from patients with TS revealed interneuron loss, metabolic stress in striatal medium spiny neurons, and enhanced inflammatory programs in microglia, providing direct human brain tissue evidence of abnormal neuron–microglia interactions (7).

The NLRP3 inflammasome is a critical molecular platform linking cellular stress to innate immune responses and is composed of NLRP3, apoptosis-associated speck-like protein containing a CARD (ASC), and pro-caspase-1. In response to pathogen-associated or damage-associated signals, NLRP3 assembles into an active inflammasome complex and activates caspase-1, thereby promoting the maturation of IL-1β and IL-18 while simultaneously cleaving GSDMD to trigger inflammatory pyroptosis (8). Within the central nervous system, microglia are major effector cells of NLRP3 signaling, and sustained NLRP3 activation can amplify neuroinflammation and compromise neuronal and synaptic function (9). Mechanistically adjacent studies have shown that excessive activation of microglial NLRP3 can enhance NMDA receptor activity through IL-1β and induce repetitive behaviors, suggesting a potential influence on excitatory synaptic transmission, although these findings were not obtained in TS models (10). Studies in animal models of TS have likewise demonstrated that suppressing striatal microglial activation and inflammatory responses can ameliorate tic-like behaviors (11). In addition, defective mitophagy may promote NLRP3 activation through the accumulation of mitochondrial reactive oxygen species and mitochondrial DNA (mtDNA) (12). A 2025 study in a rat model of TS further showed that enhancing PINK1/Parkin-mediated mitophagy suppressed NLRP3–caspase-1–GSDMD-associated pyroptotic signaling and alleviated neuroinflammation, neuronal injury, and tic-like behaviors (13). These findings suggest that a mitochondrial homeostasis–NLRP3–pyroptosis axis may contribute to TD-related neural dysfunction.

Despite these observations supporting the biological plausibility of NLRP3 involvement in TDs, its disease-specific role remains insufficiently validated. A recent study of cerebrospinal fluid from children with TS identified elevated MCP-1/CCL2 levels, providing preliminary clinical evidence of central inflammatory activity; however, NLRP3, ASC, caspase-1, and GSDMD signaling was not directly examined (14). At present, direct evidence linking NLRP3 to TDs remains largely derived from animal models, whereas pathway-level validation in pediatric patients, longitudinal biomarker studies, and targeted intervention studies are still lacking. It therefore remains unclear whether NLRP3 activation acts as a disease-driving mechanism or primarily reflects a secondary inflammatory response, and how it may further influence striatal neurotransmission, synaptic homeostasis, and CSTC circuit function. Although pharmacological strategies targeting NLRP3 and pyroptosis have entered preclinical or clinical development for several inflammatory disorders, their brain penetrance, long-term safety, and suitability for patient stratification in pediatric neurodevelopmental disorders remain uncertain (15). Accordingly, this review focuses on the NLRP3 inflammasome and systematically examines its activation and biological consequences, its potential pathological role in pediatric TDs, and the pharmacological and translational implications of NLRP3-related interventions. We further discuss the limitations of the current evidence and the major challenges to clinical translation, with the aim of proposing a testable mechanistic framework rather than an established pathogenic sequence.

## 2 Activation mechanisms and biological effects of the NLRP3 inflammasome

The NLRP3 inflammasome is a cytosolic multiprotein complex of the innate immune system that senses infection, cellular injury, and metabolic stress. Its core components include the pattern-recognition receptor NLRP3, the adaptor protein ASC, and the effector enzyme precursor pro-caspase-1. NLRP3 consists of an N-terminal pyrin domain, a central NACHT domain, and a C-terminal leucine-rich repeat domain. The NACHT domain mediates ATP binding and oligomerization and therefore provides an essential structural basis for inflammasome assembly (16). Under homeostatic conditions, NLRP3 is maintained in a relatively autoinhibited state. Upon appropriate stimulation, conformational and oligomeric rearrangements occur, enabling the recruitment of ASC and pro-caspase-1. Recent structural studies have further shown that NLRP3 activation involves conformational changes within the NACHT domain and reorganization of higher-order oligomeric structures, supporting the concept of NLRP3 as a dynamic molecular platform that converts cellular stress into inflammatory effector responses (17).

Canonical NLRP3 inflammasome activation generally involves two interconnected stages: priming and activation. The priming step is mainly initiated by Toll-like receptors, IL-1 receptors, and tumor necrosis factor receptors, which activate NF-κB signaling and promote the transcription of inflammation-related molecules, including NLRP3 and pro-IL-1β (18). In parallel, post-translational regulatory events, including ubiquitination, deubiquitination, phosphorylation, acetylation, and changes in subcellular localization, collectively establish the competence of NLRP3 for subsequent activation (19). During the activation step, extracellular ATP can trigger K⁺ efflux and other ionic fluxes through the P2X7 receptor, thereby promoting NLRP3 inflammasome activation (20). Pore-forming toxins, crystalline particles, lysosomal damage, altered Cl⁻ flux, disruption of Ca^2^⁺ homeostasis, and disassembly of the trans-Golgi network can also contribute to this process. Among these events, a reduction in intracellular K⁺ represents a major upstream signal shared by many canonical NLRP3 stimuli, although not all agonists rely on a single ionic mechanism (21). Mitochondrial injury provides another important source of activating signals. Mitochondrial reactive oxygen species, cardiolipin exposure, and the release of oxidized mitochondrial DNA can facilitate NLRP3 recruitment and assembly, with oxidized mitochondrial DNA increasingly recognized as an important danger signal linking mitochondrial homeostatic disruption to inflammasome activation (22). NLRP3 is therefore better viewed as a signaling hub that detects disturbances in intracellular homeostasis than as a receptor that directly recognizes every structurally diverse stimulus.

In response to activating signals, NLRP3 oligomerizes and recruits ASC through pyrin domain interactions. ASC subsequently polymerizes into filamentous structures or ASC specks and recruits pro-caspase-1 through CARD-mediated interactions. Recent cryo-electron microscopy studies have elucidated the structural basis of ASC filament assembly and ASC-mediated caspase-1 recruitment, providing direct structural evidence for inflammasome organization (23). Within the assembled complex, pro-caspase-1 undergoes proximity-induced dimerization and autoproteolytic processing to generate catalytically active caspase-1, which subsequently cleaves pro-IL-1β and pro-IL-18 into their mature forms, IL-1β and IL-18 (24). At the same time, caspase-1 cleaves GSDMD, releasing the pore-forming N-terminal fragment of GSDMD. This fragment inserts into the plasma membrane and forms transmembrane pores, facilitating ionic and water flux as well as the unconventional release of inflammatory mediators and, when stimulation persists, driving inflammatory pyroptotic cell death (25).

Importantly, GSDMD pore formation, cytokine release, and terminal cell lysis are not obligatorily synchronous events. Some cells can remain transiently viable while releasing IL-1β through GSDMD pores, a state commonly referred to as inflammasome hyperactivation (26). By contrast, extensive plasma membrane rupture during the terminal phase of pyroptosis, together with the release of intracellular components such as HMGB1 and nucleic acids, additionally involves membrane-disruptive mechanisms mediated by molecules including nerve injury-induced protein 1 (NINJ1) (27). Moreover, inflammatory mediators, extracellular ATP, HMGB1, and nucleic acids released from pyroptotic cells can further stimulate neighboring cells. Recent evidence also suggests that extracellular vesicles can carry functionally active GSDMD pores and propagate membrane injury, thereby contributing to inflammatory amplification (28). Overall, NLRP3 activation generates two closely interconnected but partially dissociable downstream outcomes: caspase-1-dependent maturation of IL-1β and IL-18, and GSDMD-mediated membrane pore formation and pyroptotic cell death.

Moderate and transient NLRP3 activation contributes to pathogen clearance, injury responses, and the maintenance of tissue homeostasis, whereas sustained or excessive activation can shift this protective response toward pathological inflammation. In the central nervous system, microglia are among the best-characterized effector cells of the NLRP3 inflammasome. Persistent microglial NLRP3 activation can promote the release of IL-1β, IL-18, and other inflammatory mediators, alter neuron–glia communication, and impair neuronal survival, synaptic plasticity, and neural network activity (29). Recent experimental evidence further indicates that suppressing the priming-associated transcription of Nlrp3, Casp1, and Il1b in microglia markedly reduces IL-1β release following inflammasome activation, suggesting that microglial NLRP3 responsiveness is tightly regulated by the local immune milieu (30). Sustained NLRP3 activation may therefore represent an important mechanistic link between cellular stress, neuroinflammation, and neural dysfunction, providing a biological basis for investigating its potential role in pediatric tic disorders. It should be emphasized, however, that most of the mechanistic evidence described above derives from macrophages, microglia, and models of other neurological diseases and has not yet been directly validated in pediatric TDs.

## 3 Potential pathological role of the NLRP3 inflammasome in pediatric tic disorders

### 3.1 Striatal microglial inflammation and enhancement of NLRP3-related signaling

At the level of human evidence, current studies primarily support the presence of neuroimmune abnormalities in the striatum of patients with TS, but do not establish that the NLRP3 inflammasome is specifically activated. Reanalysis of transcriptomic data from TS brain tissue has identified alterations in immune-, cytokine-, and inflammation-related pathways within the striatum (31). More recent single-nucleus transcriptomic and histological analyses of human brain tissue further revealed enhanced inflammatory transcriptional programs in microglia, metabolic stress in medium spiny neurons, and reductions in cholinergic and selected GABAergic interneuron populations within the basal ganglia of individuals with TS (7). These findings provide human brain tissue evidence of altered striatal neuroimmune states in TS; however, the studies themselves did not directly demonstrate NLRP3 inflammasome assembly or specific activation of its downstream effector pathways.

Clinical studies in children provide additional evidence that TDs may be accompanied by dynamic alterations in the immune milieu. Changes in circulating cytokines, including IL-1β, TNF-α, IL-6, and IL-4, have been reported in children and adolescents with TDs, with some markers correlating with tic severity (32). Medication-naïve children may also exhibit distinct cytokine profiles across different symptomatic stages (33), while elevated plasma CCL5 levels have been associated with the risk of TDs (34). Nevertheless, peripheral inflammatory markers cannot directly reflect the state of striatal microglia and should not be regarded as specific evidence of NLRP3 inflammasome activation. Rather, these findings mainly support the presence of a potentially systemic immune-inflammatory background in TDs.

TD/TS-related animal models have further linked local neuroinflammation to striatal dysfunction. In rats with IDPN-induced tic-like behaviors, striatal microglial activation and increased levels of inflammatory mediators such as IL-6 and TNF-α have been observed (13), together with alterations in dopaminergic markers, including tyrosine hydroxylase, PITX3, and the dopamine transporter (35). In parallel, inflammation-initiating pathways involving HDAC3/TLR4/NF-κB and TLR4/MyD88/NF-κB have been shown to decline concomitantly with reductions in microglial inflammation and tic-like behaviors (36, 37). Because TLR4/NF-κB signaling can contribute to transcriptional priming of NLRP3 and pro-IL-1β, these findings provide an upstream mechanistic context in which NLRP3 may become more readily activatable in experimental TD. However, activation of TLR4/NF-κB signaling alone should not be equated with assembly or functional activation of the NLRP3 inflammasome.

More direct disease-related evidence implicating NLRP3 still derives predominantly from IDPN-based animal models. These studies have reported alterations in striatal NLRP3, caspase-1, and gasdermin D (GSDMD)-related markers occurring alongside microglial inflammation, neuronal injury, and tic-like behaviors, with these changes being attenuated in parallel following experimental interventions (38, 39). Such findings support the possibility that NLRP3-related signaling participates in striatal inflammatory processes in experimental TD. However, changes in the expression or cleavage of individual pathway components are not, by themselves, sufficient to demonstrate the occurrence of functional pyroptosis and, in particular, cannot be extrapolated to imply GSDMD pore formation in human TDs. Current evidence therefore mainly indicates an association between NLRP3-related signaling, inflammatory changes, and behavioral phenotypes. Its disease specificity and causal role will require further validation using selective pharmacological inhibition, genetic manipulation, and direct functional assays of pyroptosis.

### 3.2 NLRP3–caspase-1–GSDMD-related signaling and potential inflammatory amplification

Evidence linking pyroptosis to TDs currently remains largely confined to molecular associations identified in animal models. Several TD/TS-related animal studies have reported alterations in NLRP3, activated caspase-1, and GSDMD-related markers, while experimental interventions were accompanied by concurrent reductions in these molecular changes, striatal neuroinflammation, neuronal injury, and tic-like behaviors (38, 39). These observations suggest that the NLRP3–caspase-1–GSDMD axis may participate in inflammatory processes in experimental TD, but they do not establish that striatal cells undergo functional pyroptosis. In particular, there is currently no direct evidence in children with TD/TS demonstrating GSDMD pore formation, characteristic pyroptotic morphological changes, or other functional indices of pyroptotic cell death.

Mechanistic evidence from non-TD neurological disease models provides a biological basis for the possibility that this pathway may amplify inflammation. In models of chronic intermittent hypoxia, microglial NLRP3–GSDMD-related signaling has been shown to promote IL-1β release and persistent neuroinflammation (40). Similarly, in neuropathic pain models, suppression of microglial NLRP3 activity has been accompanied by attenuation of pyroptosis-related changes, neuroinflammation, and behavioral abnormalities (41). More broadly, studies of pyroptosis have demonstrated that the N-terminal fragment generated by GSDMD cleavage can form membrane pores and facilitate the release of inflammatory mediators, whereas terminal plasma membrane rupture and extracellular release of intracellular danger signals, including HMGB1 and nucleic acids, are additionally regulated by membrane-injury mechanisms involving proteins such as NINJ1 (42). Together, these findings demonstrate the biological capacity of the NLRP3–caspase-1–GSDMD pathway to propagate inflammatory responses, although these functional events have not yet been directly demonstrated in TDs.

On the basis of NLRP3-, caspase-1-, and GSDMD-related molecular alterations observed in TD animal models, together with mechanistic evidence derived from non-TD systems, a testable model of inflammatory amplification can therefore be proposed. If persistent NLRP3 activation in the striatum of individuals with TDs were to progress to caspase-1 activation and functional GSDMD pore formation, the resulting cytokine release, membrane injury, and secondary danger signals could potentially enhance inflammatory responses in neighboring microglia, astrocytes, and neurons, thereby promoting the persistence of local neuroimmune activation. At present, however, this process should be regarded as a mechanistic hypothesis supported by cross-model evidence rather than as an established NLRP3–pyroptosis inflammatory amplification loop in human TDs.

### 3.3 NLRP3-related inflammation–synapse coupling and abnormal striatal information processing

Whether NLRP3-related inflammation directly influences synaptic function in TDs remains unclear because disease-specific evidence is currently lacking. The biological plausibility of such a link is derived primarily from experimental models outside the TD field. In a mouse model carrying a gain-of-function Nlrp3 variant, excessive microglial NLRP3 activation increased IL-1β release, enhanced NMDA receptor-related excitatory synaptic transmission, and induced repetitive behaviors, whereas blockade of either the IL-1 receptor or NMDA receptors partially reversed these abnormalities (10). This study established an experimental mechanistic link among microglial NLRP3 activation, IL-1β signaling, excitatory synaptic transmission, and behavioral abnormalities. However, because this was not a TS model, it does not demonstrate that the same mechanism operates in TDs. In other neurological disease models, astrocytic NLRP3-related inflammation has also been shown to affect GABA production and behavioral phenotypes (43), further suggesting that inflammasome signaling may have the capacity to modulate the balance between excitatory and inhibitory neurotransmission.

Independently of these non-TD mechanistic observations, animal models relevant to TDs support the presence of abnormalities in striatal neurotransmitter systems. Alterations in glutamate and GABA levels, as well as in their metabolism, transport, and receptor-related markers, have been reported in the striatum and related brain regions, with some of these changes being remodeled in parallel with improvement in tic-like behaviors (44–46). At the same time, a pro-inflammatory microglial state in the striatum has been associated with changes in dopaminergic markers, including tyrosine hydroxylase, PITX3, and the dopamine transporter (35). Experimental TS studies have also shown that striatal dopamine release and D2 receptor signaling are closely associated with tic expression and treatment-related effects (47). These findings indicate that neuroinflammation and neurotransmitter abnormalities can coexist in TD-related animal models; however, there is currently no direct evidence demonstrating that abnormalities in glutamatergic, GABAergic, or dopaminergic signaling are driven by NLRP3 inflammasome activation.

Accordingly, these two lines of evidence should be considered separately. Non-TD experimental studies demonstrate that NLRP3-related inflammation is capable of modulating excitatory or inhibitory synaptic function, whereas TD animal studies independently support abnormalities in striatal glutamatergic, GABAergic, and dopaminergic systems. On the basis of the biological relationship between these processes, a testable inflammation–synapse coupling hypothesis can be proposed: NLRP3-related inflammation may contribute to impaired excitatory–inhibitory integration and dopaminergic modulation in the striatum by altering neuron–glia communication and synaptic efficacy, potentially affecting how medium spiny neurons and local interneurons select and integrate cortical inputs. At present, however, this relationship remains a mechanistic hypothesis supported by cross-model evidence rather than a continuous causal pathway that has been demonstrated in TDs.

### 3.4 Abnormal striatal information processing and CSTC circuit dysfunction: a potential mechanistic link

Compared with evidence implicating NLRP3-related mechanisms, abnormalities within cortico-striato-thalamo-cortical (CSTC) circuits and related action-control networks in TDs are supported by more direct human evidence. The striatum serves as a major hub for the integration of sensory and motor information and participates in action selection and suppression of irrelevant movements through basal ganglia-related pathways. Neuroimaging and network studies have shown reduced differentiation of neural representations related to voluntary action and action inhibition in patients with TS. Children with TS also exhibit abnormal dynamic connectivity involving subcortical, sensorimotor, and higher-order control networks, some of which is associated with tic severity (48, 49). Structural connectivity studies have further identified abnormalities in corticostriatal and corticothalamic white-matter pathways, with selected connectivity measures correlating with tic severity and response to behavioral therapy (50). Lesion network mapping and deep brain stimulation connectomic studies have additionally shown that anatomically distinct lesions and stimulation targets can converge on relatively consistent tic-related functional networks involving the caudate, putamen, globus pallidus, cingulo-opercular regions, and somato-cognitive action networks (51–53). Specific functional connectivity patterns measured during tic-suppression tasks in children with TS have also been reported to predict treatment response to behavioral therapy (54). Collectively, these human studies support disrupted coordination among networks involved in action generation, sensory feedback, and cognitive control in TDs, but they do not implicate the NLRP3 inflammasome as a direct driver of these circuit abnormalities.

No study has yet established a continuous causal pathway linking abnormal NLRP3 inflammasome activation to glutamatergic, GABAergic, or dopaminergic dysregulation and, subsequently, to specific abnormalities in CSTC gating. The relationship between NLRP3 and CSTC circuit dysfunction should therefore be regarded as a hypothesis at the level of mechanistic integration. Within this framework, if NLRP3-related inflammation is indeed capable of altering neuron–glia communication, synaptic plasticity, and neurotransmitter integration in the striatum, the selection and suppression of cortical inputs by striatal circuits could theoretically be disrupted, with downstream effects on information transmission through basal ganglia–thalamocortical networks. At present, however, this represents a biologically plausible potential link rather than an established mechanism and requires validation through disease-specific neurochemical, electrophysiological, and circuit-level studies in TDs.

Taken together, current human TD/TS studies primarily support abnormalities in striatal neuroimmune states and CSTC-related network function. TD/TS animal models provide disease-context evidence for NLRP3-related signaling changes and neurotransmitter abnormalities, whereas the specific mechanisms by which NLRP3-associated inflammation can modify synaptic transmission are derived largely from non-TD experimental models. On the basis of these independent but biologically interconnected lines of evidence, we propose a working hypothesis in which NLRP3-related signaling may serve as a potential mechanistic link among striatal neuroinflammation, abnormalities in synaptic and neurotransmitter function, and neural circuit dysregulation. Importantly, no study has yet demonstrated the complete causal sequence of abnormal NLRP3 activation → neurotransmitter imbalance → disrupted striatal information processing → CSTC network dysfunction → tic expression. The value of this framework therefore lies in defining mechanistic relationships that can be experimentally tested rather than in describing an established pathogenic sequence in TDs. This conceptual framework, integrating the potential links among striatal neuroinflammation, NLRP3-related inflammatory amplification, altered synaptic and neurotransmitter processing, and CSTC circuit dysfunction, is illustrated in Figure 1. The major mechanistic propositions and their corresponding levels of evidence are summarized in Table 1.

**Figure 1:**
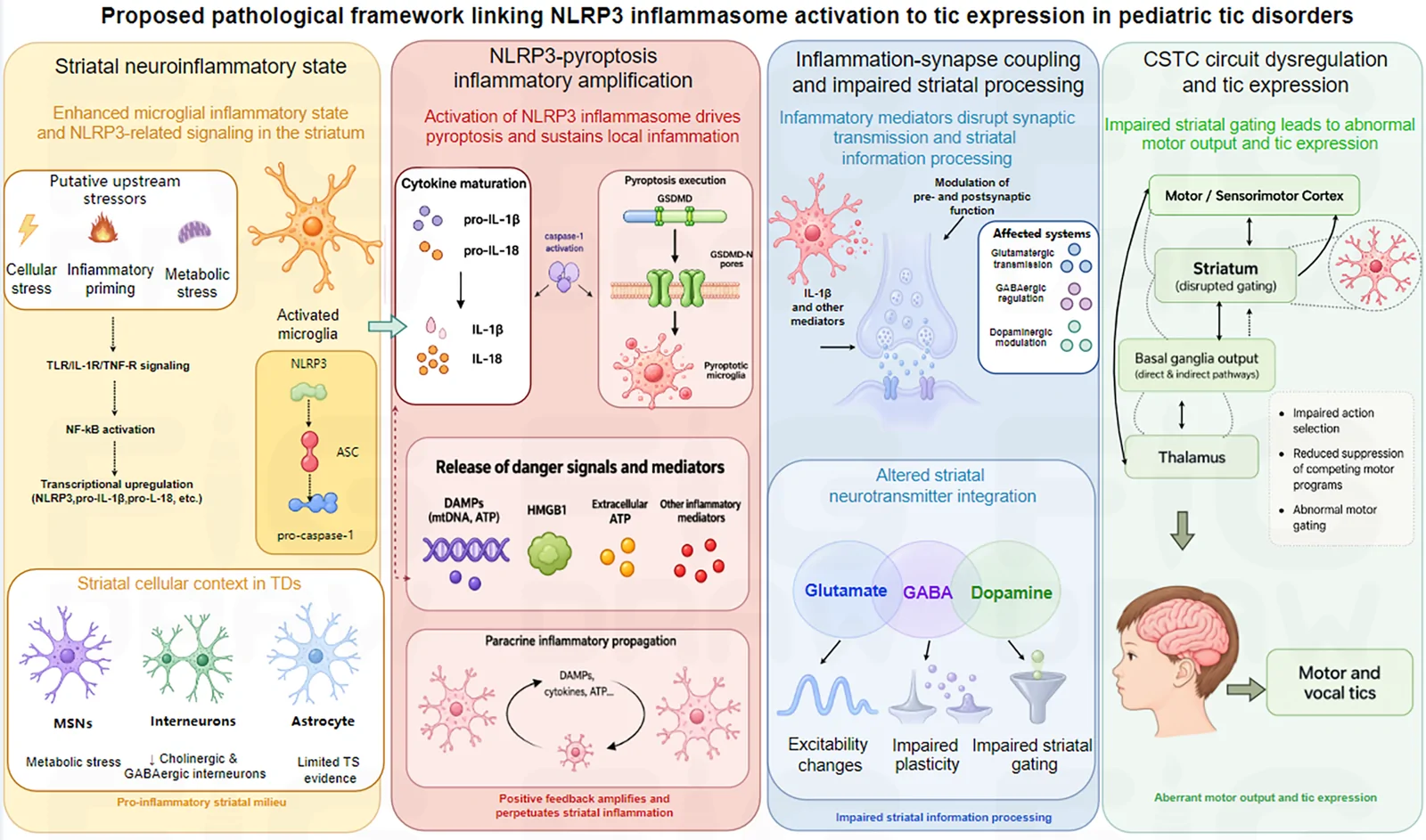
Conceptual framework of the potential involvement of NLRP3-related signaling in pediatric tic disorders. Legend: This schematic provides a conceptual integration of evidence from different experimental levels rather than a confirmed pathological sequence in pediatric TDs. Human TD/TS studies primarily support striatal neuroimmune abnormalities, microglial inflammatory changes, neurotransmitter-related alterations, and CSTC/network dysfunction, but do not directly demonstrate NLRP3 inflammasome activation or functional pyroptosis. Evidence for NLRP3-, caspase-1-, and GSDMD-related changes is derived mainly from tic-like animal models, whereas the functional mechanisms of GSDMD pore formation, pyroptosis, inflammation–synapse coupling, and NLRP3-related modulation of synaptic transmission are supported largely by non-TD experimental models. Accordingly, the depicted connections from NLRP3-related inflammation to neurotransmitter imbalance, altered striatal information processing, CSTC dysfunction, and tic expression should be interpreted as proposed mechanistic links that require disease-specific validation. The arrows and pathway relationships shown in the figure are schematic and do not denote established causal relationships in human TD/TS. In particular, GSDMD-mediated pore formation and functional pyroptosis have not been directly demonstrated in pediatric TDs. NLRP3, NOD-like receptor family pyrin domain-containing 3; ASC, apoptosis-associated speck-like protein containing a CARD; GSDMD, gasdermin D; DAMPs, damage-associated molecular patterns; CSTC, cortico-striato-thalamo-cortical; TDs, tic disorders; TS, Tourette syndrome; MSN, medium spiny neuron; mtDNA, mitochondrial DNA; ATP, adenosine triphosphate; HMGB1, high mobility group box 1; IL, interleukin; GABA, γ-aminobutyric acid.

**Table 1:** Evidence hierarchy for major NLRP3-related mechanisms in tic disorders.

Major mechanism	Human TD/TS evidence	TD/TS animal-model evidence	Non-TD mechanistic evidence	Current interpretation and references
Striatal neuroinflammation and microglial abnormalities	Yes. Human brain omics and clinical immune studies support neuroimmune abnormalities	Yes. Microglial activation and increased inflammatory mediators have been reported	—	Disease-associated neuroimmune abnormalities are relatively well supported, but they should not be equated with NLRP3-specific activation (7, 13, 31–34)
Abnormal inflammatory priming through TLR4/NF-κB and related pathways	Direct central evidence is lacking	Yes. Related signaling pathways are altered in tic-like models	Yes. These pathways can promote transcriptional priming of NLRP3 and pro-IL-1β	Represents an upstream mechanistic context for NLRP3 activation rather than direct evidence of inflammasome activation (36, 37)
Enhanced NLRP3-related signaling	No direct human evidence	Yes. Increased NLRP3-, caspase-1-, and related pathway markers have been reported	Yes. NLRP3 activation is supported in multiple neuroinflammatory models	Supported mainly by TD animal models; disease-specific causal validation remains lacking (38, 39)
Caspase-1–GSDMD-related signaling and potential inflammatory amplification	No direct evidence of functional pyroptosis	Related molecular alterations have been reported	Yes. Mechanistic studies support GSDMD pore formation and inflammatory amplification	A candidate mechanism; GSDMD pore formation and functional pyroptosis have not been directly demonstrated in TDs (38–42)
Effects of NLRP3-related inflammation on synaptic function	No direct evidence	Direct validation is lacking	Yes. NLRP3-related inflammation can affect NMDA receptor- and GABA-related functions	Represents a cross-model mechanistic inference rather than a TD-specific established mechanism (10, 43)
Glutamatergic, GABAergic, and dopaminergic abnormalities	Direct human evidence discussed in this section is limited	Substantial disease-model evidence is available	—	Neurotransmitter abnormalities are supported in TD-related models, but whether they are driven by NLRP3 remains unclear (35, 44–47)
Effects of NLRP3-related inflammation on striatal gating and CSTC network function	Human evidence supports CSTC abnormalities, but no direct link to NLRP3 has been established	No complete causal chain has been demonstrated	Mechanistic support exists for selected intermediate steps	Represents an integrative working hypothesis; the continuous NLRP3 → synapse → CSTC causal pathway remains unestablished (10, 38–54)

## 4 Pharmacological feasibility and translational evidence for NLRP3-related interventions

Given the potential involvement of NLRP3-related signaling in striatal cellular stress and neuroinflammation, its upstream regulators, the inflammasome itself, and downstream inflammatory and pyroptosis-related effectors all represent potential levels for pharmacological intervention. Current strategies can be broadly categorized into three tiers: reducing upstream drivers of aberrant inflammasome activation, directly inhibiting the NLRP3 inflammasome, and blocking downstream inflammatory or pyroptosis-related effects (Table 2). However, pharmacological tractability must be clearly distinguished from target validation in TDs. The former indicates that the relevant pathway can be pharmacologically modulated and that some candidate agents have supporting evidence for central nervous system exposure, human pharmacokinetics, or safety. By contrast, target validation requires demonstration that NLRP3 has a disease-specific functional role in TDs and that modulation of this pathway leads to improvement in disease-relevant phenotypes.

**Table 2:** Pharmacological strategies targeting NLRP3-related pathways and their translational evidence.

Intervention level	Representative strategy/drug	Major mechanism of action	Evidence base	Evidence boundary/major limitations	References
Upstream regulation of NLRP3 activation	Enhancement of PINK1/Parkin-mediated mitophagy	Promotes clearance of damaged mitochondria and reduces the accumulation of mtROS and oxidized mtDNA; Parkin may also facilitate NLRP3 degradation and thereby reduce the propensity for inflammasome assembly	TD/TS animal studies suggest that restoration of mitochondrial homeostasis can coincide with attenuation of NLRP3-related signaling, neuroinflammation, and tic-like behaviors; studies in PD and AD further support Parkin–NLRP3 and PINK1/Parkin-related regulatory mechanisms	Mitophagy has broad cellular effects, and Parkin-mediated regulation of NLRP3 is not synonymous with PINK1/Parkin-dependent mitophagy; current evidence does not establish that behavioral benefits are specifically mediated through NLRP3	(55–57)
Upstream regulation of NLRP3 activation	Modulation of ROS/oxidative stress	Reduces mitochondrial and cytosolic ROS, attenuates ROS–p38 MAPK–NLRP3-related signaling, and limits the generation or release of oxidized mtDNA	TD/TS studies indicate that ROS levels and NLRP3-related molecular markers may change in parallel; other neurobehavioral and neurodegenerative disease models further support regulation of NLRP3-associated inflammation by oxidative stress	Antioxidant interventions are not specific to NLRP3, and ROS also participates in normal cellular signaling; these findings therefore cannot independently validate a causal role for NLRP3	(58–60)
Upstream regulation of NLRP3 activation	Modulation of inflammatory priming pathways, including p38 MAPK/NF-κB	Inhibits IKK/NF-κB, PI3 K/AKT/NF-κB, or p38 MAPK signaling, thereby reducing transcriptional priming of NLRP3, pro-IL-1β, and related inflammatory molecules	TD/TS rat studies show that relevant interventions can reduce striatal NF-κB signaling and microglial activation while improving tic-like behaviors; other models further support mechanistic links between p38 MAPK and NLRP3 signaling	Most TD/TS studies involve multitarget agents or compound interventions and lack pathway-specific or rescue experiments; inhibition of NF-κB should not be equated with validation of NLRP3 as a disease target	(61–63)
Upstream regulation of NLRP3 activation	Modulation of ATP–P2X7 signaling and ionic homeostasis	Reduces ATP/P2X7-mediated K⁺ efflux and Ca^2^⁺ alterations, thereby limiting NLRP3 activation and IL-1β release; THIK-1 may additionally contribute to microglial ionic homeostasis	Studies in AD, headache models, and human brain microglia support involvement of ATP–P2X7 signaling and related ionic fluxes in NLRP3 activation	No direct TD/TS studies are available; P2X7 is broadly expressed and its effects are cell-type dependent, while the associated ionic mechanisms are complex	(64–66)
Direct targeting of the NLRP3 inflammasome	Prototypical selective NLRP3 inhibitors, such as MCC950	Acts on the NACHT domain of NLRP3 to restrict conformational changes, ATPase-associated activity, and inflammasome assembly	Structural, target-binding, and cellular studies support a direct inhibitory effect of MCC950 and related selective compounds on NLRP3	No selective NLRP3 inhibitor has yet been tested directly in TD/TS models; these compounds are currently more suitable as tools for causal validation, while pediatric brain exposure, long-term pharmacokinetics, and safety remain unknown	(17, 67)
Direct targeting of the NLRP3 inflammasome	Brain-penetrant selective NLRP3 inhibitors, including NT-0796, VEN-02XX, and MCC7840	Crosses the blood–brain barrier and suppresses central NLRP3 activation, reducing IL-1β, IL-18, and related inflammatory biomarkers	Animal studies in AD and PD, together with early-stage adult neurological studies, have provided evidence of brain exposure, target engagement, and biomarker effects	Current evidence primarily demonstrates the pharmacological feasibility of targeting central NLRP3 and should not be interpreted as target validation or clinical efficacy in pediatric TDs; long-term pediatric safety remains unknown	(68–70)
Direct targeting of the NLRP3 inflammasome	Other small-molecule NLRP3 inhibitors in clinical development, including ZYIL1 and DFV890	Selectively inhibits NLRP3 activation and downstream release of IL-1β and IL-18	Studies in healthy adults and patients with COVID-19 or systemic inflammatory disorders have provided human pharmacokinetic, safety, and pharmacodynamic data	No direct TD/TS evidence is available, and some compounds may have limited brain penetration; human findings in other diseases cannot be directly extrapolated to pediatric TDs	(71, 72)
Blockade of downstream inflammatory and pyroptosis-related effects	Caspase-1 inhibitors, such as VX-765	Reduces processing of pro-IL-1β and pro-IL-18 and GSDMD cleavage; some studies also suggest effects on mitophagy and inflammasome-related processes	Neurological injury models, including cerebral ischemia, indicate that VX-765 can attenuate pyroptosis-related changes, glial activation, and secondary neural injury	No direct pharmacological studies have been conducted in TD/TS; caspase-1 is a shared downstream effector of multiple inflammasomes and therefore lacks specificity for NLRP3	(73, 74)
Blockade of downstream inflammatory and pyroptosis-related effects	GSDMD inhibition or deficiency	Inhibits GSDMD N-terminal oligomerization, membrane insertion, and pore formation, thereby reducing membrane injury and DAMP release	GSDMD-related molecular alterations have been observed in TD animal models; Gsdmd deletion and interventions such as GI-Y1 in other disease models further support its role in regulating pyroptotic pore formation and tissue inflammation	GSDMD pore formation and functional pyroptosis have not been directly demonstrated in pediatric TDs; this approach would also not prevent NLRP3 activation itself	(75–77)
Blockade of downstream inflammatory and pyroptosis-related effects	Blockade of IL-1β/IL-1R signaling, such as anakinra	Blocks IL-1β–IL-1R1 signaling and modulates inflammatory responses in neurons and glial cells as well as potential inflammation–synapse interactions	Studies in neurological disorders such as TBI and AD support anti-neuroinflammatory and neuroprotective effects of IL-1R1 blockade	No interventional studies are available in TD/TS; this strategy does not block IL-18 or GSDMD-related effects, and prolonged inhibition may interfere with physiological synaptic regulation and motor learning	(78–80)
Blockade of downstream inflammatory and pyroptosis-related effects	IL-18 neutralization, IL-18R blockade, or IL-18-binding protein	Reduces IL-18–IL-18R signaling and IFN-γ-associated immune amplification	Models of spinal cord injury, intracerebral hemorrhage, and AD suggest that blockade of the IL-18 pathway can attenuate neuroinflammation and provide neuroprotective effects	No direct TD/TS evidence is available; disease-specific evidence for IL-18 in TDs remains limited, and the long-term immunological safety of sustained blockade in children is unknown	(81–83)

### 4.1 Modulating upstream cellular stress to limit aberrant NLRP3 activation

Reducing cellular stressors that may promote NLRP3 activation represents an intervention strategy with some support from experimental TD research. Impaired mitochondrial quality control can lead to the accumulation of damaged mitochondria, mitochondrial reactive oxygen species (mtROS), and oxidized mitochondrial DNA (mtDNA), whereas enhancement of PINK1/Parkin-mediated mitophagy facilitates the clearance of dysfunctional mitochondria. Parkin may also regulate NLRP3 stability through ubiquitination, thereby reducing the propensity for inflammasome assembly through multiple mechanisms (55–57). Consistent with this concept, reducing ROS burden may limit the generation of oxidized mtDNA and attenuate ROS–p38 MAPK–NLRP3-related signaling (58–60). In TD/TS animal studies, restoration of mitochondrial homeostasis, attenuation of NLRP3-related molecular alterations, and improvement in tic-like behaviors have been observed concurrently, providing disease-model evidence for a potential relationship between mitochondrial stress and NLRP3-associated inflammation. However, the available evidence is derived predominantly from animal studies, and these interventions simultaneously affect mitochondrial function, oxidative stress, and multiple other inflammatory pathways. It therefore remains unclear whether the observed behavioral benefits are specifically mediated through modulation of NLRP3.

Aberrant NLRP3 activation also depends on inflammatory priming and disturbances in ionic homeostasis. TD/TS animal studies have shown that modulation of TLR4/MyD88/NF-κB, PI3K/AKT/NF-κB, and p38 MAPK signaling can reduce microglial inflammatory responses and improve tic-like behaviors (61–63), suggesting that suppression of the transcriptional priming of NLRP3 and pro-IL-1β may reduce the magnitude of subsequent inflammasome responses. ATP–P2X7-mediated K⁺ efflux and alterations in intracellular Ca^2^⁺ represent another potential upstream entry point. Evidence from other neurological disease models and studies of human brain microglia supports a regulatory role for P2X7 and associated ionic fluxes in NLRP3 activation (64–66). Nevertheless, these upstream pathways participate in a broad range of physiological processes, and their modulation is not specific to NLRP3. Thus, the current advantage of upstream interventions lies in the existence of some supporting evidence from TD/TS disease models, whereas their principal limitation is the difficulty in determining whether behavioral improvement results from NLRP3 inhibition itself or from simultaneous restoration of mitochondrial function, oxidative homeostasis, and broader inflammatory networks.

### 4.2 Direct targeting of the NLRP3 inflammasome

Compared with indirect modulation of upstream stimuli, direct inhibition of NLRP3 provides a more specific strategy for determining whether NLRP3 has a disease-specific functional role in TDs. Prototypical selective inhibitors such as MCC950 act on the NACHT domain of NLRP3, stabilizing an inactive conformation and restricting ATPase-associated activity and inflammasome assembly (17, 67). From a pharmacological perspective, this approach can simultaneously influence caspase-1 activation, IL-1β and IL-18 maturation, and GSDMD-related downstream effects, making selective NLRP3 inhibition an important experimental tool for testing whether NLRP3 has a causal role in disease-related phenotypes. However, selective NLRP3 inhibitors have not yet been directly evaluated in TD/TS models, representing one of the most conspicuous gaps in the current evidence base.

The recent development of brain-penetrant NLRP3 inhibitors has further strengthened the pharmacological feasibility of targeting this pathway within the central nervous system. Candidate compounds including NT-0796, VEN-02XX, and MCC7840 have demonstrated brain exposure, evidence of NLRP3 target engagement, and changes in neuroinflammatory biomarkers in early-stage adult neurological studies or in animal models of Alzheimer's disease and Parkinson's disease (68–70), indicating that central NLRP3 is a pharmacologically accessible target. In parallel, human studies of small-molecule inhibitors such as ZYIL1 and DFV890 have provided additional pharmacokinetic, safety, and pharmacodynamic experience with systemic NLRP3 inhibition (71, 72). Collectively, these studies support the pharmacological tractability of NLRP3 in humans and, for selected compounds, within the central nervous system. However, this evidence has not yet extended to disease-specific target validation in pediatric TDs. In particular, pharmacological effects observed in adults with inflammatory or neurodegenerative disorders cannot be assumed to translate into clinical benefit in pediatric neurodevelopmental disorders.

### 4.3 Blocking downstream inflammatory and pyroptotic effects

Once NLRP3 has been activated, targeting caspase-1, GSDMD, or downstream cytokine signaling may limit inflammatory amplification at the effector level. The caspase-1 inhibitor VX-765 can reduce the processing of pro-IL-1β and pro-IL-18 as well as GSDMD cleavage, and has been shown to attenuate pyroptosis, glial activation, and secondary neural injury in other models of neurological damage (73, 74). This strategy can simultaneously interfere with cytokine maturation and the execution of pyroptosis; however, caspase-1 also serves as a common downstream effector of several inflammasomes, making its pharmacological effects broader than those of NLRP3-specific inhibition. Further downstream, pharmacological targeting of GSDMD can directly restrict N-terminal oligomerization, membrane insertion, and pyroptotic pore formation, thereby limiting membrane injury and the propagation of danger signals (75–77). Because GSDMD-related molecular alterations have been observed in TD animal models, this approach may provide a useful means of testing the functional contribution of GSDMD-mediated membrane injury and potential inflammatory amplification in TDs. Nevertheless, GSDMD inhibition would not prevent inflammasome activation itself or suppress all non-pyroptotic inflammatory outputs.

Targeting inflammatory cytokines represents an intervention point even closer to the downstream transmission of NLRP3-related inflammation into neural dysfunction. IL-1β/IL-1R1 signaling can influence neuronal and glial responses as well as inflammation–synapse coupling. Accordingly, the anti-inflammatory effects of IL-1 pathway interventions, including anakinra, in other neurological disorders provide pharmacological precedent for investigating IL-1-related inflammatory outputs in TDs (78–80). Neutralization of IL-18, blockade of IL-18R, or administration of IL-18-binding protein can likewise reduce IL-18-associated immune amplification and has shown neuroprotective effects in models of spinal cord injury, intracerebral hemorrhage, and Alzheimer's disease (81–83). Compared with IL-1β, however, disease-specific evidence implicating IL-18 in TDs remains particularly limited. More importantly, downstream blockade can selectively suppress individual pathological outputs but cannot fully shut down the broader inflammatory network driven by NLRP3. For example, inhibition of IL-1β does not suppress IL-18 signaling or GSDMD-mediated pyroptosis, whereas GSDMD inhibition does not reverse upstream inflammatory signaling associated with NLRP3 activation.

Overall, different levels of intervention are supported by distinct evidence bases and offer different forms of mechanistic insight. Upstream modulation is supported by some experimental evidence from TD/TS animal models, but lacks specificity for NLRP3. Direct NLRP3 inhibition offers greater pharmacological specificity, yet has not been directly validated in disease-relevant TD models. Downstream blockade is supported mainly by mechanistic evidence from other disease models, and its functional significance in TDs remains uncertain. At present, these strategies should therefore be viewed primarily as tools for mechanistic validation and translational investigation rather than as therapeutic approaches with established disease-specific support in pediatric TDs. Future studies should prioritize selective pharmacological targeting of NLRP3 and related pathway components, combined with genetic manipulation and rescue experiments, to establish disease-specific causal evidence in TD models. Parallel studies in pediatric patients will also be required to determine whether corresponding central inflammatory abnormalities and NLRP3-related pathway alterations are present in human TDs.

## 5 Clinical translation challenges and future directions

Although accumulating studies have begun to link the NLRP3 inflammasome with neuroinflammation, pyroptosis-related signaling, and neural dysfunction in TDs, substantial gaps remain between experimental observations and clinical translation. The most pressing question is no longer simply whether NLRP3-related molecules are altered in animal models, but whether NLRP3 constitutes a causal node in the initiation or progression of TDs. Current studies largely show that increased NLRP3-, caspase-1-, and GSDMD-related signaling coexists with tic-like behaviors, and that multitarget interventions can simultaneously attenuate inflammatory changes and behavioral abnormalities. Such findings, however, are insufficient to establish NLRP3 as a necessary disease driver. Future studies should therefore incorporate Nlrp3- or Gsdmd-specific genetic manipulation, microglia-selective interventions, pathway rescue experiments, and temporally resolved analyses across different disease stages. These approaches will be important for determining whether aberrant NLRP3 activation precedes tic development, contributes to symptom maintenance, or primarily reflects a secondary inflammatory response during the disease course.

Translation from animal models to pediatric patients represents a second major challenge. Direct evidence implicating NLRP3 currently derives predominantly from experimental models such as IDPN-induced tic-like phenotypes, whereas clinical studies in children have focused mainly on peripheral cytokines, cerebrospinal fluid inflammatory markers, and neuroimaging abnormalities. Changes in circulating IL-1β, IL-18, or other inflammatory mediators cannot be assumed to reflect NLRP3 activation in striatal microglia. Future studies will therefore require multidimensional assessment frameworks capable of integrating peripheral immune status, central neuroinflammation, and neural circuit function. Combining measurements of NLRP3, ASC, caspase-1, GSDMD, and related inflammatory mediators with cerebrospinal fluid biomarkers, functional or molecular imaging, tic severity, and longitudinal disease trajectories may provide a more reliable basis for determining the relationship between NLRP3 activity and disease state. Single-cell and spatial omics approaches may further identify the specific cell populations, striatal microenvironments, and disease stages in which NLRP3-related abnormalities occur, thereby narrowing the gap between current mechanistic models and the biological reality of pediatric TDs.

The marked clinical heterogeneity of TDs also suggests that, even if NLRP3 contributes to disease mechanisms in some patients, the magnitude and relevance of this contribution are unlikely to be uniform. Infection exposure, immune status, comorbidity patterns, symptom fluctuations, and neural circuit abnormalities may jointly shape the inflammatory burden of individual patients. Future studies should therefore first determine whether a subgroup of patients exists in whom prominent neuroinflammatory features coincide with NLRP3-related abnormalities, rather than assuming that NLRP3-targeted strategies would be applicable to all patients or to any predefined clinical subtype of TDs. A stratification framework integrating NLRP3-related molecular markers, inflammatory biomarkers in biological fluids, clinical phenotypes, and neuroimaging features could help clarify whether NLRP3 activity is associated with disease progression or treatment response and whether it has potential value for biomarker development and patient stratification.

At the same time, safety considerations in children must remain central to translational development. NLRP3 contributes to normal host defense and tissue homeostasis, while childhood is a period of ongoing immune maturation, synaptic remodeling, and neural circuit development. Prolonged or broadly suppressive inhibition of inflammasome signaling could therefore increase susceptibility to infection or interfere with normal neuroimmune functions. Future therapeutic studies should avoid assuming that complete suppression of NLRP3 will necessarily confer clinical benefit and should instead first define its disease-specific contribution and the appropriate degree and timing of intervention. Brain penetrance, cellular selectivity, therapeutic window, age-dependent dosing, and long-term neurodevelopmental safety will all require careful evaluation before pediatric clinical testing can be considered.

Overall, further development of NLRP3-related interventions in TDs will require a continuous evidence chain extending from disease-specific causal validation and the establishment of central biomarkers to patient stratification and rigorous safety assessment. Only after it has been established whether a biologically distinct subgroup of patients exhibits NLRP3-dependent disease mechanisms will the clinical value of NLRP3 as a precision therapeutic target be meaningfully assessable.
